# Seasonal effects on multiparous dairy cow behavior in early lactation

**DOI:** 10.3168/jdsc.2022-0358

**Published:** 2023-09-28

**Authors:** I.M. Toledo, L.T. Casarotto, G.E. Dahl

**Affiliations:** 1 Institute of Food and Agricultural Sciences (IFAS) Extension, University of Florida, Gainesville, FL 32608; 2 Department of Animal Sciences, University of Florida, Gainesville, FL 32608

## Abstract

Controlled studies have shown that heat stress abatement positively influences health, productivity, behavior, and reproductive performance of dairy cows during all stages of the lactation cycle. Based on previous findings, the present study focused on a better understanding of how seasonal changes affect the behavior of multiparous lactating dairy cows kept in typical free-stall housing with the objective to aid in the management of lactating cows exposed to variable environmental conditions. Automated monitoring devices (Nedap, the Netherlands) were used to assess behavioral activity of mature Holstein dairy cows during the “hot season” (HS; n = 19; July, August, and September) and the “cool season” (CS; n = 15; December, January, and February) under normal management conditions. Cows received a leg tag to measure daily lying time, and number of steps and standing bouts, and a neck tag to measure eating and rumination time. All cows were housed in sand-bedded freestall barns equipped with cooling systems (soakers and fans). Behavior, milk production and milk components were recorded for the first 9 wk of lactation after calving. Average temperature-humidity index (THI) was 78.2 ± 0.4 (± standard error) in the HS and 54.4 ± 0.2 in the CS. Fat-corrected milk yield was greater in the CS compared with HS during the first 5 wk of lactation. Milk protein percentage was lower in CS during the first 2 wk of lactation. In contrast with HS, milk fat percentage was greater in the CS. Compared with CS, overall, during HS cows spent less time eating, lying down, and tended to spend less time ruminating. In addition, exposure to high THI resulted in increases in standing bouts, and overall standing time in HS relative to CS. No differences in number of steps were observed between HS and CS. In summary, exposure to high THI during lactation seems to negatively affect the behavior and consequently the daily time budget of lactating Holstein cows, even under housing conditions with active cooling. A better understanding on how different seasons affect the daily time budget of lactating dairy cows may contribute to the development of more effective management strategies to decrease the negative effects of heat exposure.

## Highlights

• Exposure of Holstein lactating cows to high THI affects behavior and daily time budget, even in housing conditions with active cooling.
• During the summer, cows spend less time eating, ruminating, lying, and standing, and have more standing bouts.
• Seasonal changes affect milk production and composition of multiparous dairy cows.

## Graphical Abstract

**Figure:**
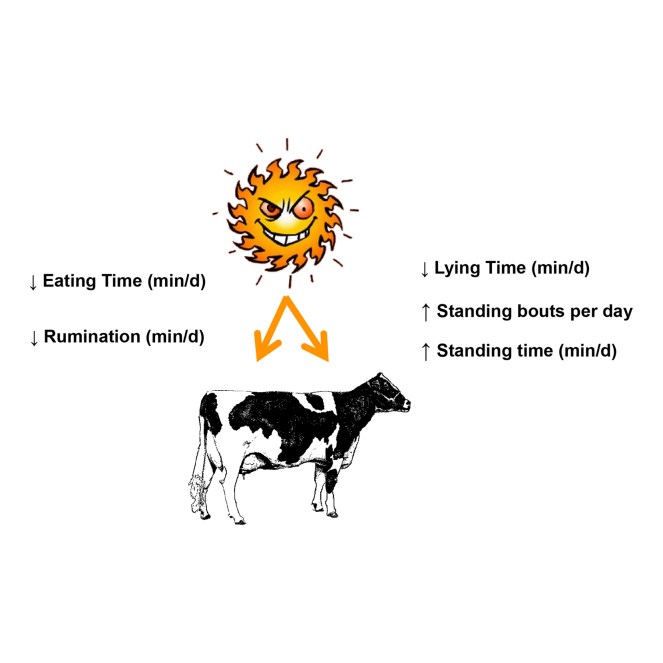
**Summary** Exposure of early lactating dairy cows to a high temperature-humidity index (THI) affects the daily time budget, even in facilities equipped with active cooling systems. During the hottest months of the year, cows spend less time eating, ruminating, and lying, and spend more time standing and have more standing bouts per day. Environmental management strategies associated with increases in temperature during seasonal changes should be considered to attain optimal performance during lactation.

Lactating dairy cows produce a large amount of metabolic heat, which, in combination with exposure to high temperatures, results in excessive heat load and decreased ability of the cow to dissipate excess body heat. Physiological and behavioral adaptations are strategies adopted by lactating dairy cows to cope with the increased heat load associated with seasonal changes in temperature. Collier et al. (2012) has shown that physiological changes and decreases in production of lactating dairy cows begin to be adversely affected in conditions of a temperature-humidity index (**THI**) as low as 68, although a recent study suggests decreases in daily milk production with THI as low as 64 (Ji et al., 2020). Such THI measurements are prevalent in even more temperate regions, let alone subtropical locales such as Florida.

Controlled studies have extensively documented that exposure to heat stress negatively affects health, productivity, behavior, and reproductive performance of dairy cows during all stages of the lactation cycle. Lactating cows exposed to heat stress experience decreased milk production and immune responses, lowered reproductive performance, and increased incidence of postpartum diseases (Hahn, 1999; Kadzere et al., 2002; Collier et al., 2006). In addition, milk components such as fat and protein are also affected by exposure to high temperatures (Rodriquez et al., 1985; Bernabucci et al., 2015; Cowley et al., 2015). Furthermore, exposure to heat stress during the dry period not only negatively affects the dam's milk production in the subsequent lactation (Tao et al., 2011; Thompson et al., 2014), but also exerts persistent negative effects on the offspring during their entire productive life (Laporta et al., 2020).

Behavioral strategies adopted by lactating cows to adapt to environmental changes include increased standing time (Nordlund et al., 2019), decreased activity and movement (West et al., 2003), reduced rumination time (Soriani et al., 2013), decreased DMI (Wheelock et al., 2010), and modification of drinking and eating habits. Although physiological and behavioral adaptations are necessary, they affect the performance and production of lactating dairy cows, which often result in financial losses (St-Pierre et al., 2003; Ferreira et al., 2016).

To alleviate the negative effects of exposure to high temperatures, heat abatement technologies such as shade, fans, and soakers are commonly used for lactating cows on US dairies (Spiers et al., 2018). However, despite the efforts to reduce the negative effect associated with exposure to high temperatures, in regions of hot and humid climate, it is common to observe seasonal effects on behavioral activities, performance, and production of lactating dairy cows. Thus, even when cooling systems are provided, heat stress abatement may not fully overcome the impacts of a seasonal heat load, and cows may thus exhibit behavioral changes to deal with the heat stress.

The hypothesis of this study is that seasonal changes will affect the behavior and production of multiparous lactating dairy cows housed in freestall facilities and exposed to active cooling. Our objectives were to better understand how seasonal changes affect the behavior of multiparous lactating dairy cows to aid producers manage cows exposed to variable environmental conditions.

This study was conducted at the University of Florida Dairy Unit (Hague, FL) during the summer months (July, August, and September) of 2020 and the winter months (December, January, and February) of 2020 and 2021. Animal handling and experimental procedures were approved by the Institutional Animal Care and Use Committee at the University of Florida.

Thirty-four lactating multiparous Holstein cows, with similar genetic potential (evaluated by the PTA records), were enrolled in the study during the “hot season” (**HS**; n = 19; July, August, and September) and the “cool season” (**CS**; n = 15; December, January, and February) shortly after parturition (d 3 after calving). Cows remained in the study for 60 d, and all animals enrolled completed the study. Behavioral activity and milk production were recorded during the first 9 wk of lactation. During both seasons, all cows were housed in a sand-bedded freestall barn and were provided with the shade of the barn in addition to water soakers (Rain Bird Manufacturing, Glendale, CA) over the feed line, and fans (J&D Manufacturing, Eau Claire, WI). Fans ran continuously over the stalls when temperatures exceeded 20.0°C and the water soakers turned on automatically for 1 min at 5-min intervals when ambient temperatures exceeded 21.1°C. Across both seasons, stocking density varied between 90% and 100%. Lights were on for 14 h/d at an intensity of 150 lx throughout the study, therefore all cows were under the same photoperiodic conditions regardless of season.

During both seasons, all cows were fed a TMR formulated to meet the nutrient requirements for lactating cows (NRC, 2001). Feed was pushed up several times each day. Free access to water was provided. Cows were milked twice daily at approximately 0700 and 1900 h according to the standard operating procedures of the University of Florida Dairy Unit.

The THI was assessed during the study period and was calculated based on the equation recommended by Dikmen et al. (2008): THI = (1.8 × T + 32) − [(0.55 − 0.0055 × RH) × (1.8 × T − 26)], where T = ambient temperature (°C) and RH = relative humidity (%).

Milk yields were retrieved from AfiFarm Dairy Herd Management Software (Afimilk Ltd., Kibbutz, Afikim, Israel) and recorded until 63 DIM. Percentages of milk fat and protein were measured at each milking by an AfiLab real-time milk analyzer (SAE Afikim, Kibbutz Afikim, Israel) until 63 DIM. The AfiLab milk analyzer is based on the optical characteristics of light scattering off matter such as milk fat and protein and has been validated to reflect measurements from near-infrared spectroscopy-based estimates (Kaniyamattam and De Vries, 2014).

Automated monitoring devices (Nedap, the Netherlands) were used to document the behavioral activity of cows during the study periods. Upon enrollment, all cows were equipped with a “Smarttag Leg” (434 MHz) and a “Smarttag Neck” (FER4). Behavior recordings started 2 d after enrollment and were documented every 15-min period. The leg tag measured daily lying time, number of steps, and standing bouts, and the neck tag quantified eating and rumination times. The “Nedap Smarttags” use a G-sensor, which uses acceleration as a measure of movement in a particular direction, based on a 3-dimensional accelerometer. The tags distinguish forward and backward, left and right, and up and down movements. The Nedap Smarttags have previously been validated to assess behavioral activity levels in dairy cattle (Van Erp-Van deKooij et al., 2016). The Nedap software provides data that include the daily average for each behavioral activity. Data extracted from the Nedap software are ready to be used in further statistical analysis.

All statistical analyses were performed in SAS (version 9.4, SAS Institute Inc.). Data were tested for covariance (Levene's test) and normality of distribution was tested by evaluating Shapiro-Wilk statistics using the univariate procedure. The THI was averaged per day and analyzed with generalized linear mixed models using the PROC MIXED procedure of SAS. All of the other variables were submitted to repeated-measures variance analysis using a mixed model (PROC MIXED procedure). The model included the fixed effect of treatment, time (i.e., week), and the interaction between treatment and time. The cow within treatment was used as a random effect, and PTA was used as a covariate. All statistical comparisons were performed by Tukey-Kramer testing. Significance was set at *P* ≤ 0.05 and tendencies were declared at 0.10 ≤ *P* > 0.05.

It has been documented that multiparous lactating dairy cows start to experience heat stress and milk production losses when THI is as low as 68 (Collier et al., 2012) or even 64 (Ji et al., 2020). In the present study, the estimation of environmental thermal variations due to changes in season on animal performance was determined by THI measurements. Temperature-humidity index measurements differed between HS and CS (78.2 ± 0.2 vs. 54.4 ± 0.4; *P* < 0.01), indicating that HS and CS cows were exposed to distinct thermal environmental conditions. Observation of a 78.2 THI during the HS confirms that lactating cows were exposed to significant heat stress during the study period, and potentially subjected to production losses, whereas CS cows were not.

In agreement with what has been reported before in cows exposed to high THI, in the present study we observed a season by week interaction with decreases in milk production during the first 5 wk of lactation during HS compared with CS. Relative to HS, CS cows produced more FCM during the first 5 wk of lactation (46.2 ± 1.8 vs. 41.9 ± 1.6 kg; *P* < 0.01). Seasonal milk component differences were also observed. In contrast with HS, fat percentage was greater in the CS (4.05 ± 0.63 vs. 3.83 ± 0.53 kg; *P* < 0.01). Moreover, treatment by time interaction was detected regarding protein percentage. Relative to HS, CS cows had a lower percentage of protein during the first 2 wk of lactation (2.90 ± 0.05 vs. 2.98 ± 0.04 kg; *P* < 0.01). In contrast with the present results, Bernabucci et al. (2015) documented that during the summer, milk of Holstein cows has 9.9% less protein than in spring. Cowley et al. (2015) shown detrimental effects of exposure to heat stress on milk protein composition. However, there were no residual effects of heat stress on production of protein that continued after the period of exposure to heat stress. Despite the efforts to investigate the effects of high temperatures on milk protein, there are still discrepancies among studies.

The greater fat percentage observed during CS agrees with previous studies that have documented significant decreases in milk fat percentage during the summer compared with the winter months. Rodriquez et al. (1985) conducted a study involving nearly 23,000 observations in Florida dairy farms and reported the relationship between milk composition and environmental temperature. Likewise, in the present study, as temperature increased, milk fat concentrations dropped.

In the present study, seasonal changes significantly affected most of the recorded behavioral activities, despite the access to active cooling at all times. Adaptation of behavioral activities have been associated with changes in health status (Dittrich et al., 2019), management (Grant and Albright, 2001), and environmental conditions (Nordlund et al., 2019). In addition, behavioral activity has been widely used as an indicator of welfare and comfort (Haley et al., 2000; Müller and Schrader, 2003) and to investigate production parameters of farm animals (Phillips and Rind, 2001).

In the present study, we observed the seasonal effects of increased temperatures on behavioral activity. To the best of our knowledge, this study is the first of its kind to evaluate the effects of seasonal heat stress on behavioral activity of multiparous lactating dairy cows in a humid subtropical climate. A treatment effect was observed for eating time. During HS, cows spent less time eating (134 ± 13.1 vs. 199 ± 14.2 min/d; *P* < 0.01) and tended to spend less time ruminating (558 ± 25.8 vs. 629 ± 28.2 min/d; *P* = 0.07) in comparison with CS cows (Figures 1a and 1b). In addition, eating time measurements showed a significant season by week interaction (*P* < 0.01 and *P* < 0.10; Figure 1a); thus, due to the week by season interaction, no significant differences in eating time between seasons were observed during wk 1, 2, and 3. Recent studies report similar results regarding eating and rumination time adaptations in situations of heat stress. Ramón-Moragues et al. (2021) observed that when dairy cows are exposed to high THI values, they reduce eating and rumination times. Corazzin et al. (2021) used Italian Holstein-Friesian cows to show that feeding behavior is affected by heat stress, and the use of cooling strategies is associated with increases in both rumination and eating times, similar to those observed in the present study. Even though in the present study feed intake was not measured, previous studies have reported the relationship between decreases in feed intake as an adaptation strategy to maintain homeostasis (Collier et al., 2019).

**Figure 1:**
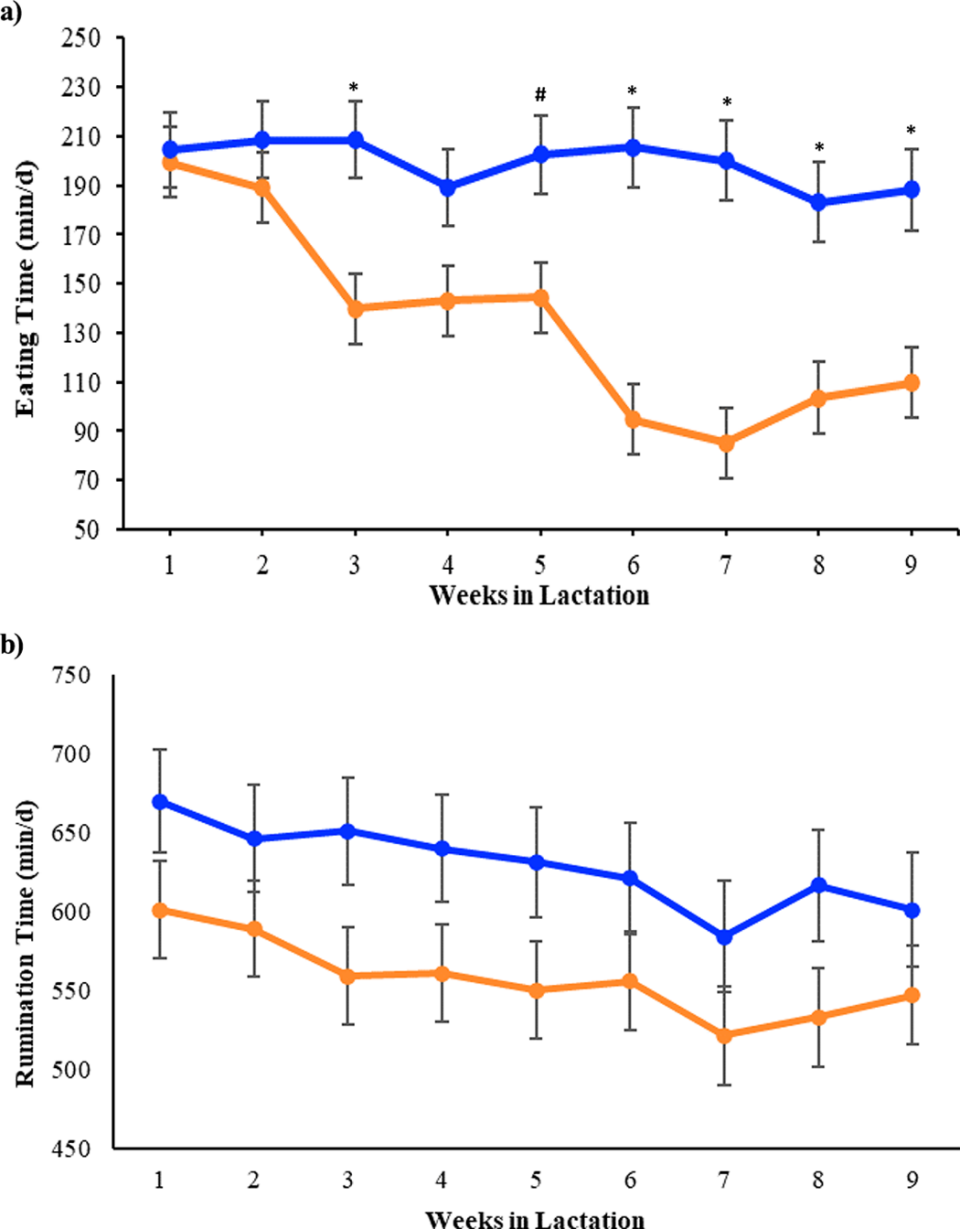
Eating (a) and rumination (b) time (min/d) from wk 1 to 9 of lactation. During both hot (HS; orange) and cool (CS; blue) seasons, Holstein dairy cows were kept in a freestall barn with shade and were cooled by soakers and fans during the entire study period. A treatment effect was observed, where HS cows spent less time eating (*P* < 0.01) and tended to spend less time ruminating (*P* = 0.07) compared with CS cows. Season by week significance is represented by * and # in panel a: *P < 0.01; #*P* < 0.10. Data are presented as LSM ± SEM.

Although changes in rumination time are usually associated with diet characteristics, additional studies have documented negative associations between exposure to high temperatures and rumination time in both primiparous and multiparous lactating cows (Kadzere et al., 2002; Soriani et al., 2013). Moreover, reports have shown that when mature cows experience decreases in the rumination time, a delay in passage of the rumen digesta may occur, resulting in a reduction of the ruminal capacity to hold more feed. Therefore, in situations of heat stress, decreases in rumination time are associated with decreases in both DMI and production capability (Warren et al., 1974; Church, 1988; Moallem et al., 2010). Even though in the present study we did not measure DMI, the decrease in eating and rumination times during exposure to hot temperatures may be considered as behavioral adaptations to heat stress. Since consumption of feed and forage digestion create large amounts of metabolic heat production, as temperature rises and decreases in heat dissipation capability occur, cows decrease their feed intake as a strategy to maintain homeostasis. Reduced DMI is also the likely explanation for the lower milk yield during HS relative to CS.

The amount of time cows spent lying down was affected seasonally, even with active cooling. Overall, HS cows had significant reduction in lying time relative to CS (717 ± 21.1 vs. 814 ± 23.9 min/d; *P* < 0.01; Figure 2a). A season by week interaction was also significant for lying time (*P* < 0.05 and *P* < 0.10; Figure 2a). Thus, no significant differences in lying time between seasons were observed during wk 3, 4, 5, and 8. In addition, increased standing time was significant during HS compared with CS (720 ± 21.3 vs. 626 ± 24.0 min/d; *P* < 0.01; Figure 2b). Season by week interaction was also significant for standing time (*P* = 0.03; *P* < 0.10; Figure 2b), which represents no significance of treatment on wk 3, 4, 5, 8, and 9. These results agree with previous reports where, in attempts to increase heat loss, lactating cows reduced lying time and increased standing times in conditions of heat stress (Overton et al., 2002; Cook et al., 2007; Nordlund et al., 2019). Furthermore, exposure to environmental heat resulted in increases in standing bouts (15 ± 0.7 vs. 12 ± 0.7 stands/d; *P* < 0.01; Figure 2c), showed a significant season by week interaction (*P* < 0.01; Figure 2c), and demonstrated no significance of treatment on wk 1, 2, 4, 7, 8, and 9. This behavioral adaptation was probably used as a strategy to cope with the high temperatures during the summer months to increase dissipation of heat. No differences in the number of steps (3,172 ± 138.7 vs. 3,288 ± 156.7 steps/d; *P* = 0.58) were observed between HS and CS cows, which is probably related to the fact that fresh animals used in the study were kept in the same pen during different seasons and had to walk the same distance to the milking parlor; thus, no direct impact of seasonal changes was observed on this behavioral activity parameter.

**Figure 2:**
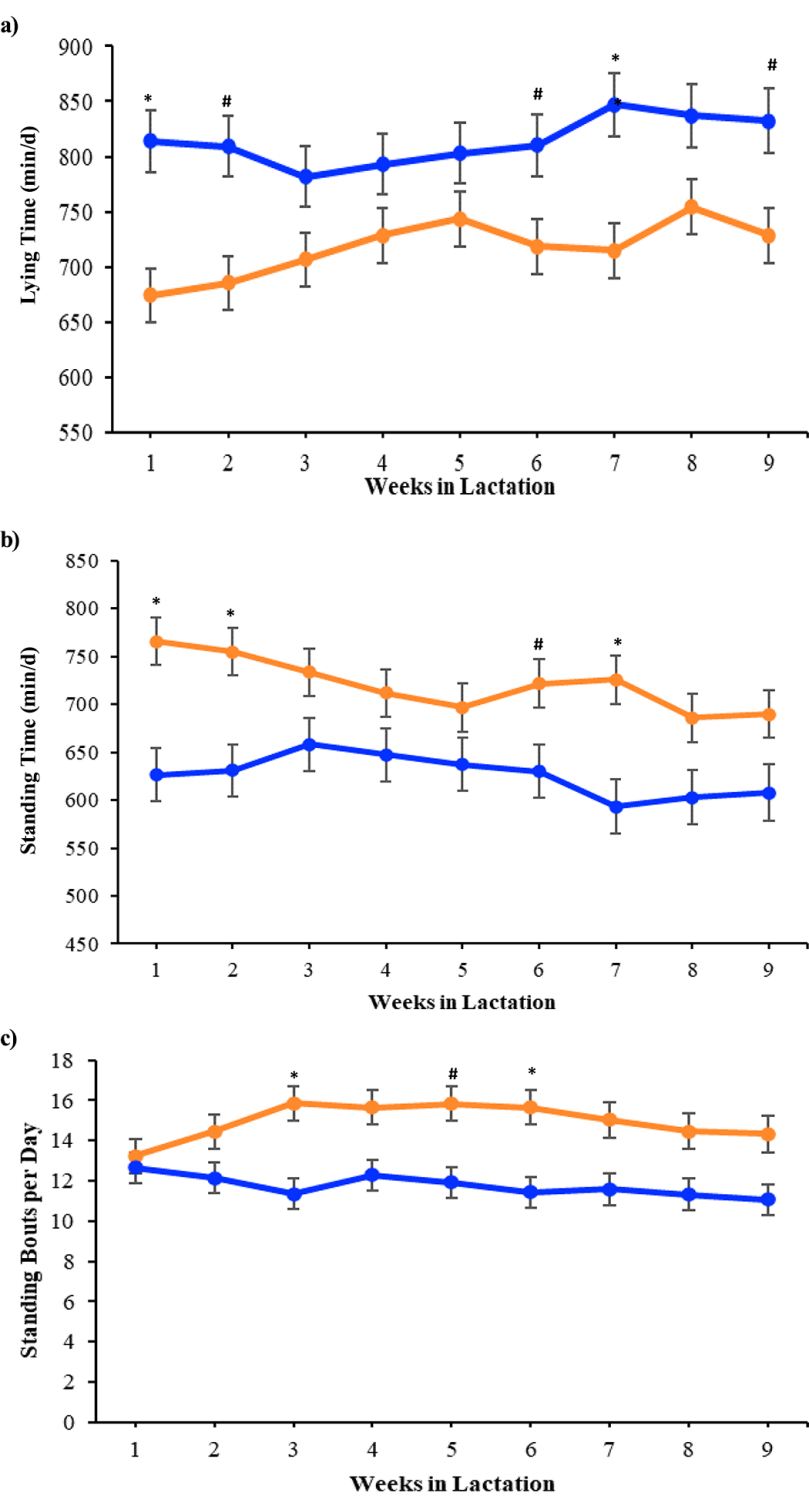
Lying time (a; min/d), standing time (b; min/d), and standing bouts (c; bouts/d) from wk 1 to 9 of lactation. During both hot (HS; orange) and cool (CS; blue) seasons, cows were kept in a freestall barn with shade and were cooled by soakers and fans during the entire study period. A treatment effect was observed. During HS, cows had significantly decreased lying time (*P* < 0.01) and increased standing time (*P* < 0.01) and standing bouts (*P* < 0.01). Season by week significance is represented by * and # in all panels (*P < 0.05; #*P* < 0.10). Data are presented as LSM ± SEM.

In conclusion, exposure of lactating dairy cows to high THI during the first 60 DIM negatively affected the behavior and consequent daily time budget of lactating Holstein cows, even under normal housing conditions with active cooling. Due to exposure to high temperatures, overall, HS cows spent less time eating, ruminating, and lying down. To increase heat dissipation, HS cows spent more time standing and had more standing bouts compared with CS cows. Environmental management strategies associated with increases in temperature during seasonal changes should be considered to attain optimal performance during lactation.
